# Anxiety and Depression in Autistic College Students: The Freshman Survey Results

**DOI:** 10.7759/cureus.35820

**Published:** 2023-03-06

**Authors:** Kashia A Rosenau, Emily Hotez, Priyanka Fernandes, Christopher Gomez, Kevin Eagan, Lindsay Shea, Alice Kuo

**Affiliations:** 1 Medicine, University of California, Los Angeles, Los Angeles, USA; 2 Clinical Child Psychology, University of Kansas, Lawrence, USA; 3 Education and Information Studies, University of California, Los Angeles, Los Angeles, USA; 4 AJ Drexel Autism Institute, Drexel University, Philadelphia, USA; 5 Pediatrics, University of California, Los Angeles, Los Angeles, USA

**Keywords:** depression, college, emerging adults, anxiety, autism

## Abstract

Objective

Mental health among undergraduate students is a growing concern in higher education, but relatively little is known about the mental health of autistic college students. In order to better understand the unique needs of this population, the present study examines whether demographic and psychosocial correlates of anxiety and depression differ in autistic first-year college students and their non-autistic peers.

Methods

Secondary data analysis was conducted utilizing population-weighted data of full-time college students in their first year attending four-year colleges and universities in 2016, 2018, and 2019. Autistic and non-autistic students who self-identified as having frequent anxiety or depression were compared in terms of demographic characteristics, physical and emotional health, and academic aspirations and achievement.

Results

The majority of first-year students with frequent anxiety or depression in this sample tended to be white and had parents who completed a bachelor's degree or went to graduate school, with higher rates of male students in the autistic group. While autistic college freshmen with frequent anxiety or depression self-report lower overall quality of physical health (below average or lowest 10% reported by 57.3% vs. 37.1%) and higher rates of learning disabilities (25.3% vs. 4.6%) and psychological disorders (62.3% vs. 29.3%), these students also tend to outperform their non-autistic peers on standardized academic testing.

Conclusion

As autistic students are investing in themselves through their education and future careers, practitioners and researchers alike should be investing in accessible physical and mental health services in order to help set autistic students up for success in college and beyond.

## Introduction

The first year of college is a pronounced transition that involves many potential stressors for students as they simultaneously adjust to their newfound responsibilities and independence. It is a time that allows more freedom for some, who gain more independence in college, and it spikes an accumulation of numerous personal and familial responsibilities for others, who are now balancing college life in addition to their previous obligation. Given these substantial life changes, it is not surprising that symptoms of anxiety and depression are commonly experienced by first-time college students [1].

Prevalence rates of mental health disorders in college students

Previous research has shown university students demonstrated patterns of poor mental health and depressed mood [2]. In fact, the World Health Organization (WHO) reports that half of the disease burden amongst young adults in the United States (US) is due to disorders related to mental health. Similarly, an international study of mental health in college students, the WHO World Mental Health - International College Student Project (WMH-ICS), screened for depression, anxiety, and substance use/abuse in 13,984 emerging adults from 19 colleges across eight countries. The international 12-month occurrence of mental health disorders was 31%, with depression being the most commonly reported (18.5%), while the occurrence rate in the United States was slightly lower at 27% [2]. This means that more than a quarter of all college students who participated in the study in the United States have likely experienced poor mental health in the last year.

The rates of mental health disorders reported by the WMH-ICS are consistent with reports from larger studies of college students conducted solely in the United States. For example, the Healthy Minds study sampled 43,210 undergraduate students across 72 universities and found that 18.2% of students screened positive for depression, while 10.1% screened positive for anxiety, and 7.8% of students reported suicidal ideation. Overall 34.4% of students in the Healthy Minds Study reported experiencing some form of mental health problem in the last year [3].

Another large-scale study of college students in the United States, the 2019 National College Health Assessment from the American College Health Association (ACHA), surveyed 54,497 undergraduate students from 98 colleges and reported that 88% of college students had felt overwhelmed by all they had to do in the last 12 months. Although feeling overwhelmed may be anticipated during college, more severe signs of mental health were also reported, with 72% of students reporting that they felt "very sad", while 46% reported "feeling so depressed that it was difficult to function", and 66% reported "feeling overwhelming anxiety" [4]. Understanding more about students who are most at risk may help educators and mental health professionals become more proactive in providing additional support for those who need it most.

The National Autism Indicators Report (NAIR) on Health and Health Care reports that anxiety and depression are more common in autistic transition-age youth (14-25; TAY) than non-autistic TAY [1]. Autistic college students are disproportionately impacted by mental health challenges, with students on the autism spectrum more frequently endorsing depressive symptoms, feeling overwhelmed, and having higher rates of lifetime psychiatric diagnoses than their non-autistic peers [5]. Similar trends exist in adulthood (18+), as approximately one-third (29%) of autistic adults experience anxiety, and over a quarter (26%) experience depression [1].

Demographic correlates of anxiety and depression in college students

Mental health challenges in college students are often associated with specific demographics [6,7]. A small body of literature has begun to examine whether these associations are also present in autistic students. Previous research indicates that non-Hispanic white autistic individuals report higher rates of both anxiety and ADHD compared to other racial and ethnic groups. This finding may be explained by health inequities related to racial and ethnic disparities in access to mental health services [1]. Additionally, in both autistic and non-autistic populations, female college freshmen are more likely to self-report experiencing anxiety and depression than males [4,5]. Although less frequently examined in the autism literature, academic performance and psychosocial variables also meaningfully predict mental health status in the general college population, with lower grades in high school [4], less social support [8,9], and lower socioeconomic status [10] each associated with poorer mental health outcomes.

It is evident from previous research that the prevalence of mental health disorders, particularly anxiety and depression, interact with social determinants such as gender, ethnicity, sexuality, and socioeconomic status [11]. In order to fully understand the risk factors for poor mental health in college students, it is essential to look at mental health from the intersection of other relevant variables, such as physical health, academic aspirations, and additional social characteristics.

Present study

The present study analyzed the Cooperative Institutional Research Program's (CIRP) Freshman Survey by the Higher Education Research Institute (HERI) based at the University of California, Los Angeles [12]. In order to better understand and address anxiety and depression among autistic college students, it is important to also look at the intersection of mental health with other relevant variables. The present study compared autistic students who self-reported frequent anxiety or depression to their non-autistic peers who also reported frequent anxiety or depression and examined the differences between these two groups regarding 1) demographic characteristics, 2) physical and emotional health and disabilities, and 3) academic performance and aspirations.

## Materials and methods

The CIRP Freshman Survey is a nationally representative survey that began in 1996 as part of a longitudinal study. The freshman survey is interested in assessing various first-year college students' pre-college experiences, including academic preparation, political and social views, career, educational, and lifelong goals, and expectations for college. Since 1996, more than 15 million students attending more than 1,110 institutions have responded to the survey. 

Survey data was utilized from the 2016, 2018, and 2019 administrations because these administrations included questions about diagnosed disabilities and chronic illnesses. While a question related to autism was added to the survey in 2012, a specific anxiety question was not added until 2016, and all questions included in the current analyses were not included yearly until 2019 (previously even years, 2016, 2018). The question that inquired about diagnosed disabilities was "Do you have any of the following disabilities or medical conditions?". Students were presented with several options, including "autism spectrum disorder", "physical disability", and "learning disability"; an "other" option was also available. Anxiety and depression were self-reported, "In the past year, how often have you" a) "felt depressed" or b) "felt anxious," and respondents were instructed to mark either "not at all," "occasionally," or "frequently." 

Analyses compared the responses from students that self-identified as autistic to those of their counterparts who did not disclose a diagnosis of autism. Population weights for the students' sex, race or ethnicity, and various characteristics of their institutions (e.g., control public, private), type (university, four-year college), and selectivity level (average SAT Math and Verbal scores)) were created for each survey cohort. The weights for the sample were created to represent all first-time, full-time, and first-year students beginning at four-year nonprofit colleges and universities in the United States in 2016, 2018, and 2019. The complete study methods and data analysis procedures are described in Fernandes et al. [5].

## Results

The total unweighted sample included 2252 first-time, full-time autistic students who reported frequent anxiety and depression compared to 187,337 first-time, full-time non-autistic students who also reported frequent anxiety or depression. With the population weights applied, the sample of autistic students who reported frequent anxiety or depression represented 18,335 (.4%) first-time, full-time students, and the weighted comparison group represented 1,602,326 (35%) non-autistic first-time, full-time college students who reported frequent anxiety or depression. Meanwhile, 2,999,129 students (65%) did not report experiencing frequent anxiety or depression. Given the large sample size and high probability of obtaining statistically significant findings, significant findings are reported only if they reflect a group difference of 2% or greater.

As shown in Table 1, more than two-thirds of non-autistic students reporting frequent anxiety or depression were female (69.2%), whereas females comprised less than half (39.9%) of the autistic group. There was a lower percentage of freshmen who identified as Asian, Black, or Latino in the autistic group (5.7%, 5.6%, and 3.6, respectively) than in the non-autistic group (8.5%, 9.2%, and 9.9%). A higher percentage of autistic students identified as white or multi-racial (64.6% and 19.6%, respectively) compared to the non-autistic group (55.7% and 15.6%). There were lower rates of autistic students whose parents have a high school diploma or less compared to their non-autistic peers (14.3% versus 19.1%) and more autistic students with parents who attended graduate school compared to parents of students in the non-autistic group (37.2% versus 31.6%).

**Table 1:** Demographic information of first-time full-time college students stratified by self-reported frequent anxiety or depression

Variables	Frequent anxiety or depression (%)	Autism and frequent anxiety or depression (%)	p-value
Gender			
Female	69.20%	39.90%	< .001
Age			
Under 18	0.90%	1.40%	< .001
18	71.10%	63.70%	.001
19	25.80%	21.70%	.006
20 or older	1.30%	3.10%	.001
Race/ethnicity			
Native American / American Indian	0.20%	0.10%	< .001
Asian	8.50%	5.70%	< .001
Black	9.20%	5.60%	.010
Latino	9.90%	3.60%	< .001
White	55.70%	64.60%	< .001
Other	0.80%	0.80%	< .001
Multi-racial	15.60%	19.60%	< .001
Parent/guardian education			
High school diploma or less	19.10%	14.30%	< .001
Bachelor's degree	32.20%	32.60%	< .001
Some college	17.10%	15.90%	< .001
Graduate school	31.60%	37.20%	< .001
Income			
Under $30,000	17.80%	16.50%	< .001
$30,000 - $59,999	15.70%	12.60%	< .001
$60,000 - $99,999	22.80%	25.70%	< .001
$100,000 - $149,999	20.70%	22.50%	< .001
$150,000-$249,999	13.60%	14.40%	< .001
$250,000 and higher	9.50%	8.30%	< .001

Table 2 presents differences in physical health and disabilities. Autistic students more commonly endorsed the below average and lowest 10% of physical health categories (42.0% and 15.3%, respectively) compared to their non-autistic peers (30.7% and 6.4%) and above average and average physical health were more commonly reported by non-autistic students (16.4% and 39.6%, respectively) than autistic students (10.4% and 25.8%). Similarly, autistic students more commonly endorsed the below-average and lowest 10% emotional health categories (22.9% and 4.3%, respectively) compared to their non-autistic peers (14.3% and 1.9%). The highest 10% and above average categories of emotional health were more commonly endorsed by non-autistic students (11.8% and 27.8%, respectively) than autistic students (8.9% and 19.7%). Frequent anxiety was endorsed by a vast majority of freshmen in both the autistic (91.9%) and non-autistic (93.9%) groups; however, frequent depression was much more commonly reported by autistic students (55.5%) than non-autistic students (37.1%). Learning disabilities, attention deficit hyperactivity disorder (ADHD), physical disabilities, chronic illness, psychological disorders, and other disabilities were all reported at higher rates by autistic freshmen than their non-autistic peers.

**Table 2:** Health characteristics of first-time full-time college students stratified by self-reported frequent anxiety or depression ADHD - attention deficit hyperactivity disorder

Variables	Frequent anxiety or depression (%)	Autism and frequent anxiety or depression (%)	p-value
Physical health			
Highest 10%	6.90%	6.50%	< .001
Above average	16.40%	10.40%	< .001
Average	39.60%	25.80%	< .001
Below average	30.70%	42.00%	< .001
Lowest 10%	6.40%	15.30%	< .001
Emotional health			
Highest 10%	11.80%	8.90%	< .001
Above average	27.80%	19.70%	< .001
Average	44.30%	44.20%	< .001
Below average	14.30%	22.90%	< .001
Lowest 10%	1.90%	4.30%	< .001
Frequent feelings of (in past year)			
Depression	37.10%	55.50%	< .001
Anxiety	93.90%	91.90%	< .001
Being overwhelmed by all they had to do	72.50%	70.70%	< .001
Disabilities or chronic illnesses			
Learning disability	4.60%	25.30%	< .001
ADHD	9.30%	40.10%	< .001
Physical disability	6.10%	21.10%	< .001
Chronic illness	3.70%	13.10%	< .001
Psychological disorder	29.30%	62.30%	< .001
Other disability	8.30%	23.70%	< .001

As shown in Table 3, autistic freshmen were more likely to report a bachelor's degree or a Ph.D. as their top degree aspiration (31.2% and 16.0%, respectively) than non-autistic freshmen (24.2% and 12.5%). Non-autistic freshmen were more likely to report a master's degree or a medical/health-related degree as their top degree aspirations (38.8% and 11.3%, respectively) compared to autistic freshmen (33.0% and 5.0%). Autistic freshmen more commonly reported B+ and B average grades in high school (22.7% and 18.4%, respectively) compared to non-autistic students (19.0% and 15.6%). A+/A and A- grades in high school were more commonly reported by non-autistic freshmen (29.4% and 27.9%, respectively) than autistic freshmen (25.5% and 23.8%). Autistic students reported higher mean scores on the Scholastic Aptitude Test (SAT), both on the math (M=612.28(113.13) autistic students and M=593.53(107.30) non-autistic students) and verbal (M=631.98(103.53) autistic students and M=611.53(94.11) non-autistic students) sections.

**Table 3:** Academic characteristics of first-time full-time college students stratified by self-reported frequent anxiety or depression

Variables	Frequent anxiety or depression (%)	Autism and frequent anxiety or depression (%)	p-value
Degree aspirations			
None	0.50%	1.50%	< .001
Vocational certificate	0.10%	0.90%	< .001
Associate degree	0.70%	0.90%	< .001
Bachelor's degree	24.20%	31.20%	< .001
Master's degree	38.80%	33.00%	< .001
Law degree	4.50%	3.40%	< .001
Medical/health-related degree	11.30%	5.00%	< .001
Doctor of Philosophy	12.50%	16.00%	< .001
Professional doctorate	6.80%	6.10%	< .001
Other	0.70%	2.30%	< .001
Average grade in high school			
A or A+	29.40%	25.50%	< .001
A-	27.90%	23.80%	< .001
B+	19.00%	22.70%	< .001
B	15.60%	18.40%	< .001
B-	5.10%	5.20%	< .001
C+	2.00%	2.60%	< .001
C	1.00%	1.70%	< .001
Standardized test scores (Mean, SD)			
American College Testing (ACT) Composite	25.47 (6.18)	25.96 (6.48)	< .001
Scholastic Aptitude Test (SAT) Math	593.53 (107.30)	612.28 (113.13)	< .001
SAT Verbal	611.53 (94.11)	631.98 (103.53)	< .001
Number of college applications submitted (Mean, SD)	5.52 (2.92)	5.15 (2.92)	< .001

Finally, self-reported academic and social characteristics are reported in Table 4. Academic ability (20.5% vs. 18.2%, p<.001), drive to achieve (35.8% vs. 33.5%, p<.001), leadership ability (25.4% vs. 22.0%, p<.001), mathematical ability (15.0% vs. 10.5%, p<.001), intellectual self-confidence (21.6% vs. 15.0%, p<.001), social self-confidence (18.8% vs. 11.4%, p<.001), spirituality (13.0% vs. 11.7%, p<.001), and risk-taking (14.3% vs. 12.6%, p<.001) all had significantly higher reports from the comparison group than from students with anxiety and depression; however, artistic ability (10.0% vs. 6.8%, p<.001), creativity (19.9% vs. 15.0%, p<.001), understanding of others (31.8% vs. 35.7%, p<.001), writing ability (15.7% vs. 10.9%, p<.001), and compassion (33.7% vs. 24.0%, p<.001) were all significantly higher in the mental health group than in the comparison group. The two latent variables of academic self-concept and pluralistic orientation were comparable between the two groups; however, the reported means of the latent construct of social self-concept were significantly higher in the comparison group, M = 51.60 (8.70) compared to M = 47.19 (9.48), p<.001, than in students reporting frequent anxiety or depression.

**Table 4:** Self-reported academic and social characteristics of first-time full-time college students stratified by self-reported frequent anxiety or depression

Variables	Comparison group (%)	Frequent anxiety or depression (%)	p-value
Self-Rated, Highest 10%			
Academic ability	20.50%	18.20%	< .001
Artistic ability	6.80%	10.00%	< .001
Creativity	15.00%	19.90%	< .001
Drive to achieve	35.80%	33.50%	< .001
Leadership ability	25.40%	22.00%	< .001
Mathematical ability	15.00%	10.50%	< .001
Public speaking ability	13.30%	13.20%	< .001
Intellectual self-confidence	21.60%	15.00%	< .001
Social self-confidence	18.80%	11.40%	< .001
Spirituality	13.00%	11.70%	< .001
Understanding of others	25.70%	31.80%	< .001
Writing ability	10.90%	15.70%	< .001
Compassion	24.00%	33.70%	< .001
Computer programming skills	2.8	2	< .001
Risk-taking	14.30%	12.60%	< .001
Academic self-concept latent construct, mean (SD)	50.60 (8.86)	48.68 (9.29)	< .001
Social self-concept latent construct, mean (SD)	51.60 (8.70)	47.19 (9.48)	< .001
Pluralistic orientation latent construct, mean (SD)	49.68 (9.32)	50.73 (9.05)	< .001

## Discussion

The present study highlights the unique challenges in autistic and non-autistic college freshmen who experience frequent anxiety or depression. Although autistic students with mental health challenges self-report lower overall quality of health and higher rates of learning disabilities and psychological disorders, autistic first-year college students also tend to outperform their non-autistic peers on standardized academic testing.

Physical and emotional health

Autistic students in this sample tend to have significantly higher rates of depression, learning disabilities, ADHD, physical disabilities, chronic illnesses, and psychological disorders than their non-autistic peers. Below-average self-reported physical and emotional health was consistently endorsed by autistic college freshmen at higher rates than their non-autistic peers who are also experiencing frequent anxiety or depression, both in the present study and previous reports [1,5]. These findings underscore the need for the integration of physical health and mental health services for autistic TAY in order to identify students who are at risk for mental health challenges early, provide coordinated care and transition planning, and make a concerted effort to enact policies that reduce stigmatizing individuals and reduce minority group related stressors [1,5].

The public health model of disability seeks to address both individual determinants of health, and population-level supports to increase the quality of care for all people [13]. An increased focus on transition planning from high school to post-secondary education could help establish a support system for autistic college freshmen - and all incoming college freshmen with mental health challenges - to help with coping skills and goal setting, as well as stress and anxiety [14].

Academic performance

Autistic college students who report frequent anxiety or depression scored significantly higher on the SAT standardized achievement test than their peers who also report frequent anxiety or depression but do not identify as autistic, yet autistic freshmen with frequent anxiety or depression reported lower average grades in high school than their non-autistic peers with similar mental health challenges. These findings warrant additional considerations for the evaluation of academic performance in neurodivergent learners with frequent anxiety or depression - for example, examining the discrepancies between ability and achievement [15]. Previous reports on transition planning show improvements in academic-related skills, goal setting, and awareness of the availability of resources on college campuses for autistic individuals who received transition planning assistance [14].

Limitations and future directions

Several limitations in data collection constrain the current findings and warrant future research. First, analyses in the current study drew from secondary data, which constrained the variables that could be included to those included in the original survey. Second, anxiety and depression were measured using a single question. The use of a single question to measure anxiety and depression constrains the implications that can be drawn from the results. In addition, the sample did not represent the experiences or perspectives of students enrolled at two-year institutions or those enrolled in college part-time. As a result, results from the current study are not generalizable to all autistic or college-student experiences. While the utilization of a nationally representative sample is a major strength of the present study, the anxiety question was not added to the survey until 2016 and thus limited the sample size. Finally, analyses used data that was collected through self-report. It is possible that participants may have misrepresented themselves as being autistic or decided to not disclose their diagnosis in the study.

In line with the public health approach to physical and mental health care for autistic individuals, the results of the present study emphasize the unique experiences of autistic college freshmen while also examining the larger issue of mental health challenges in freshmen college students [14,15]. Previous research suggests that increased transition planning improves students' early college success and lowers stress [16,17]. Educational resources geared towards primary care providers and student health centers can assist in the transition planning process for TAY to reduce stress during the transition from high school to post-secondary education [18].

## Conclusions

The current findings highlight the need for physicians, educators, and student health centers to collaborate to improve students' mental and physical health status, especially autistic freshmen who report frequent anxiety and depression. An integrated nationwide approach utilizing the public health model of disability to address both population-level issues and individual challenges is needed to effectively address mental health concerns in college student populations. As autistic and non-autistic students alike are investing in themselves through their education and future careers, practitioners and researchers similarly should be investing in accessible physical and mental health services in order to reduce disparities for autistic populations and set students up for success in college and beyond.
